# Publisher Correction: Epigenetic landscape reveals MECOM as an endothelial lineage regulator

**DOI:** 10.1038/s41467-023-38708-x

**Published:** 2023-05-18

**Authors:** Jie Lv, Shu Meng, Qilin Gu, Rongbin Zheng, Xinlei Gao, Jun-dae Kim, Min Chen, Bo Xia, Yihan Zuo, Sen Zhu, Dongyu Zhao, Yanqiang Li, Guangyu Wang, Xin Wang, Qingshu Meng, Qi Cao, John P. Cooke, Longhou Fang, Kaifu Chen, Lili Zhang

**Affiliations:** 1 Center for Bioinformatics and Computational Biology, Department of Cardiovascular Sciences, Houston Methodist Research Institute, Houston, TX USA; 2 Center for Cardiovascular Regeneration, Department of Cardiovascular Sciences, Houston Methodist Research Institute, Houston, TX USA; 3 Basic and Translational Research Division, Department of Cardiology, Boston Children’s Hospital, Boston, MA 02115 USA; 4 Department of Pediatrics, Harvard Medical School, Boston, MA 02115 USA; 5 Department of Urology, Northwestern University Feinberg School of Medicine, Chicago, IL USA; 6 Robert H. Lurie Comprehensive Cancer Center, Northwestern University Feinberg School of Medicine, Chicago, IL USA

**Keywords:** Differentiation, Epigenomics, Angiogenesis

Correction to: *Nature Communications* 10.1038/s41467-023-38002-w, published online 25 April 2023

The original version of this Article contained an error in Figure 3f. The data for “MECOM g-2” was omitted. This has been corrected in the PDF and HTML versions of the Article.

