# Supplementary material for: Epigenetic landscape reveals MECOM as an endothelial lineage regulator
Source: Nat Commun. 2023 Apr 25;14:2390. doi: 10.1038/s41467-023-38002-w (PMC10130150; doi:10.1038/s41467-023-38002-w)
Supplement: Supplementary file 1 — Supplementary Information [file 41467_2023_38002_MOESM1_ESM.pdf]

Supplementary Figures and Figure Legends

(S1-S9)

Epigenetic Landscape Reveals MECOM As an Endothelial Lineage Regulator

Jie Lv[1, *], Shu Meng[2, *], Qilin Gu[2, *], Rongbin Zheng[3, 4], Xinlei Gao[1, 2, 3, 4], Jun-dae Kim[2], Min Chen[3, 4], Bo Xia[1], Yihan Zuo[3, 4], Sen Zhu[1], Dongyu Zhao[1, 2, 3, 4], Yanqiang Li[1, 2, 3, 4], Guangyu Wang[1, 2, 3, 4], Xin Wang[1, 2, 3, 4], Qingshu Meng[5, 6], Qi Cao[5, 6], John P. Cooke[2, ^], Longhou Fang[2, ^], Kaifu Chen[1, 2, 3, 4, ^], Lili Zhang[2, 3, 4 ^]

[1]Center for Bioinformatics and Computational Biology, Department of Cardiovascular Sciences, Houston Methodist Research Institute, Houston, TX, USA
[2] Center for Cardiovascular Regeneration, Department of Cardiovascular Sciences, Houston Methodist Research Institute, Houston, TX, USA
[3] Basic and Translational Research Division, Department of Cardiology, Boston Children's Hospital, Boston, MA 02115, USA
[4] Department of Pediatrics, Harvard Medical School, Boston, MA 02115, USA
[5] Department of Urology, Northwestern University Feinberg School of Medicine, Chicago, IL, USA
[6] Robert H. Lurie Comprehensive Cancer Center, Northwestern University Feinberg School of Medicine, Chicago, IL, USA

* These authors contributed equally

^ Corresponding:
Lili Zhang, Lili.Zhang@childrens.harvard.edu
Kaifu Chen, kaifu.chen@childrens.harvard.edu
Longhou Fang, lhfang@houstonmethodist.org
John P. Cooke, jpcooke@houstonmethodist.org

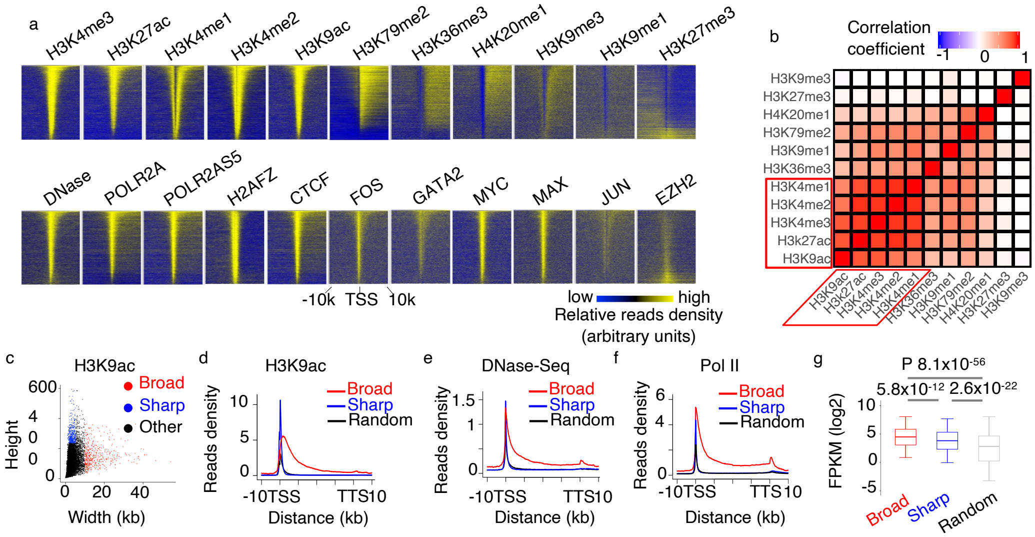

Figure S1 Super active chromatin domains signify cell identity genes in EC (a) Heat maps to show density of individual chromatin marks (panels) at each base pair (column) around each TSS (row). **(b)** Spearman correlation coefficient for each pair of active histone modifications. **(c)** H3K9ac peak height plotted against width. **(d-f**) Average signal intensity of **(d)** H3K9ac modification **(e)** DNase accessibility and **(f)** RNA polymerase II binding in three epigenetic patterns. **(g)** Expression levels of genes associated with three epigenetic patterns. Box plots indicate median (middle line), 25th, 75th percentile (box) and 5th and 95th percentile (whiskers). n=1000 genes in each group (**g**). P values are determined by two-tailed Wilcoxon test implemented in R v4.0.2 (**g**).

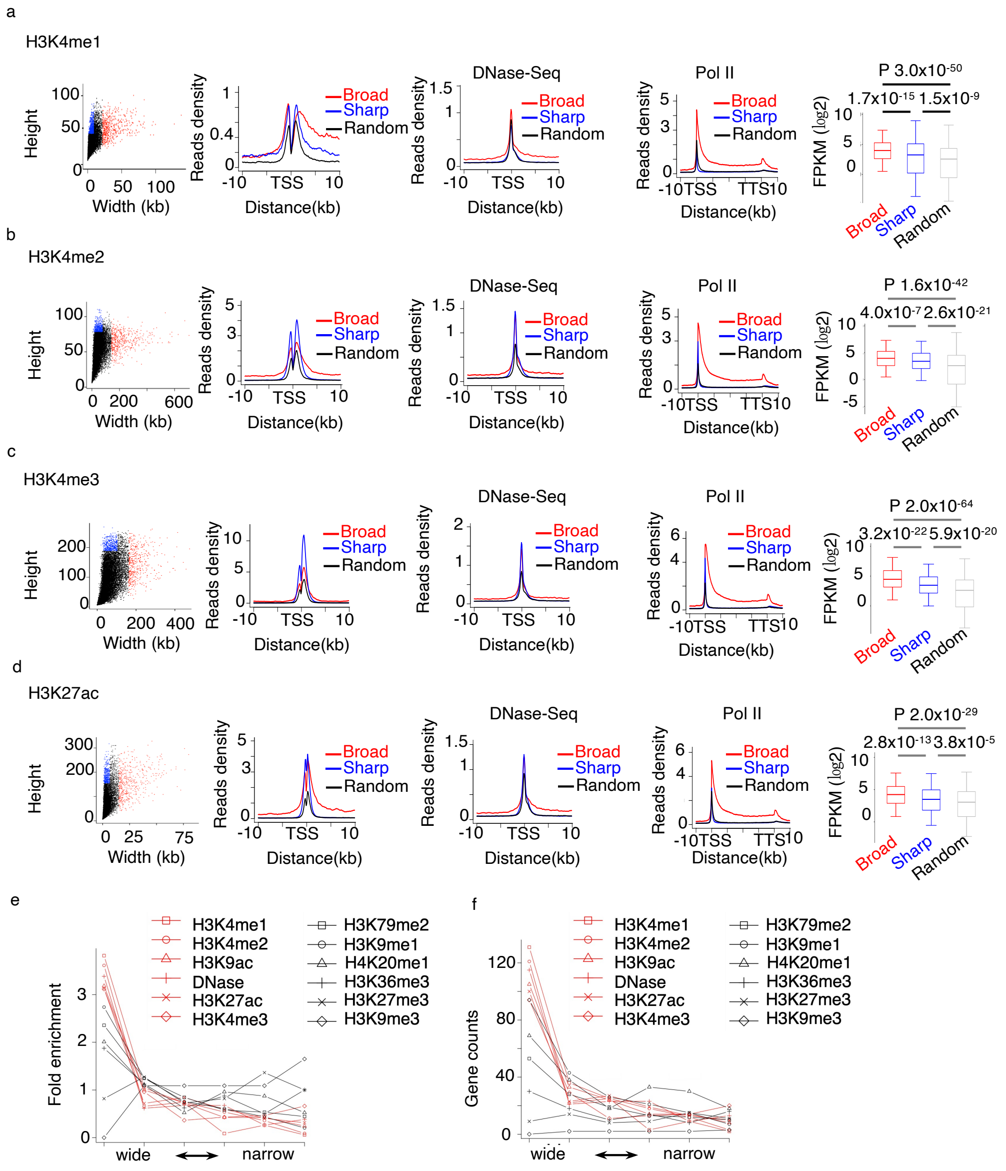

Figure S2 Broad enrichment of histone marks in HUVEC at super active domains (a-d) for H3K4me1 **(a)**, H3K4me2 **(b)**, H3K4me3 **(c)** and H3K27ac **(d)**, plot of peak height against width, average signal intensity of histone mark, DNase accessibility and RNA polymerase II binding, as well as expression levels of genes associated with three epigenetic patterns are shown. Fold enrichment **(e)** and gene counts **(f)** of EC identity regulators divided into six bins based on the width of enrichment peaks for each chromatin mark. Box plots indicate median (middle line), 25th, 75th percentile (box) and 5th and 95th percentile (whiskers). n=1000 genes in each group (**a-d**). P values are determined by two-tailed Wilcoxon test implemented in R v4.0.2 (**a-d**).

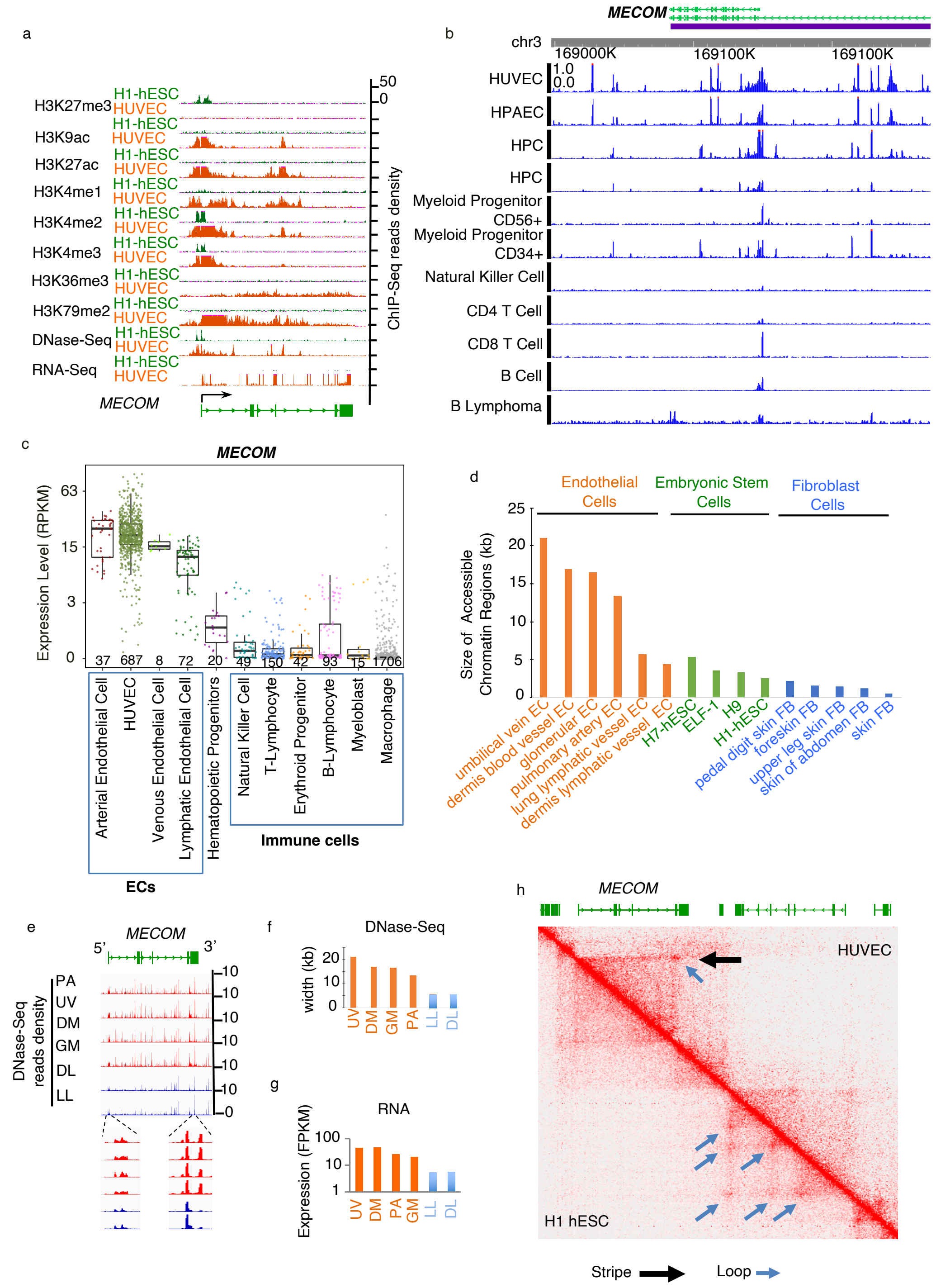

Figure S3 Epigenetic landscape reveals MECOM to be an EC lineage regulator. (a) Signal density of individual chromatin marks, DNase-Seq, and RNA-seq at the *MECOM* locus in H1-hESC and HUVEC. **(b)** The representative WashU epigenome tracks of DNase-Seq profiles showed the chromatin accessibility at the *MECOM* gene promoter across different cell types. **(c)** The gene expression of *MECOM* in RNA-Seq profiles of different cell types including EC subtypes, HPC, and immune cells. Each dot in the box plot represents one RNA-Seq sample obtained from ARCHS[4] project. Box plots indicate

median (middle line), 25th, 75th percentile (box) and 5th and 95th percentile (whiskers). Sample numbers analyzed in each group are indicated in the figure. **(d)** DNase peak width at *MECOM* locus across EC, ESC and FB subtypes. **(e)** Genome tracks of DNase-Seq and RNA-Seq signal at *MECOM* locus across 6 EC subtypes. (**f**) Width of DNase-Seq enrichment peaks at *MECOM* locus across 6 EC subtypes. (**g**) *MECOM* expression level across 6 EC subtypes. PA, pulmonary artery EC; UV, umbilical vein EC; DM, dermis microvascular blood EC; GM, glomerular microvascular EC; DL, dermis microvascular lymphatic EC; LL, lung lymphatic EC. (**h**) Two-dimensional heat map showing the chromosomal contact frequency in HUVEC and H1 hESC around *MECOM* locus. Blue arrows indicate loops. Black arrow indicates stripe.

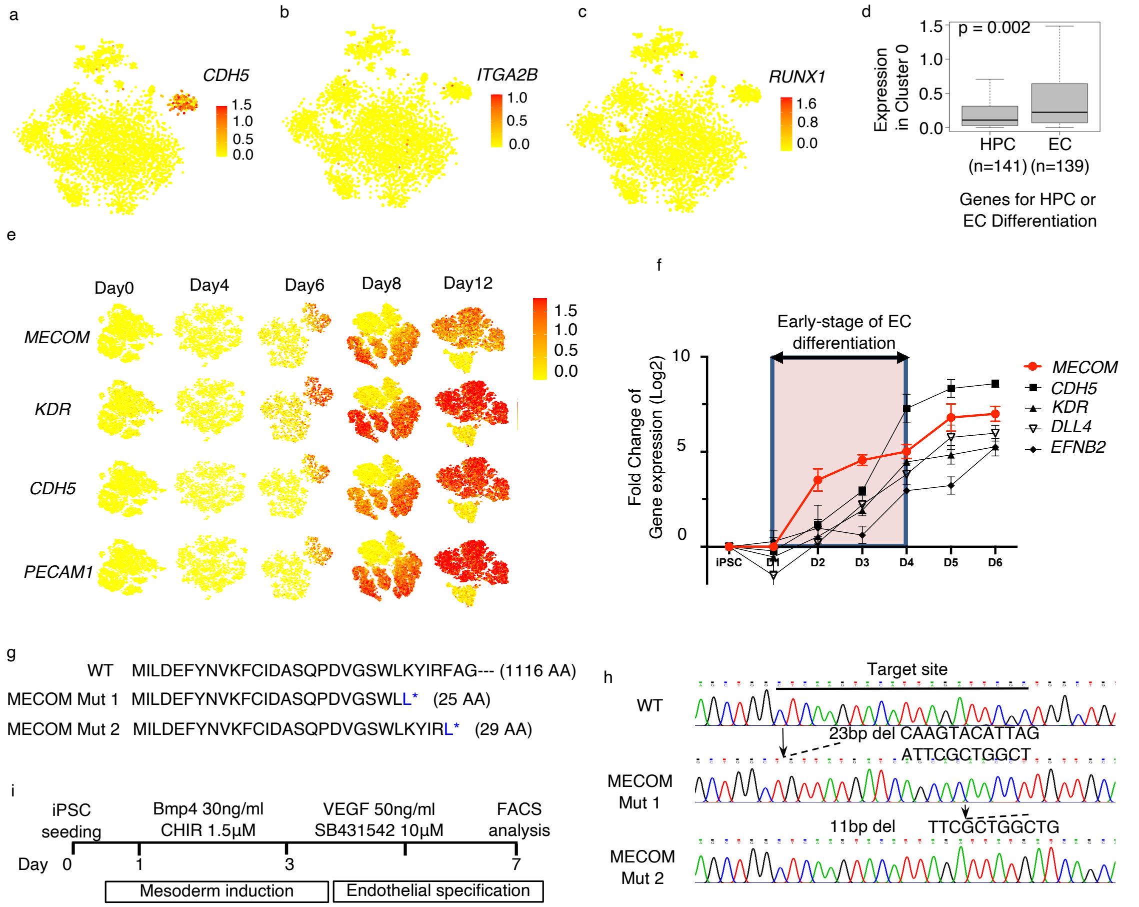

Figure S4 MECOM is required for EC differentiation and phenotypes. (a-c) Expression profiles of *CDH5* **(a)**, *ITGA2B* **(b)**, and *RUNX1* **(c)** across combined cells from days 8 and 12 of EC differentiation. The color represents the normalized expression level by Seurat package in R. **(d)** The expression level of genes related to HPC and EC differentiation in Cluster 0. Box plots indicate median (middle line), 25th, 75th percentile (box) and 5th and 95th percentile (whiskers). Gene numbers analyzed in each group are indicated in the figure. The p-value was calculated by two-tailed Wilcox test. **(e)** expression of individual genes (rows) at individual days (columns) of EC differentiation. **(f)** Relative

gene expression levels of *MECOM* and other EC marker genes during EC differentiation. Data are presented as mean values +/- SD. n=3 biologically independent samples. **(g)** WT MECOM protein sequence and putative mutant sequences in iPSC. **(h)** Alignment of DNA sequence between human WT and mutant MECOM showing the position and size of deleted fragments. **(i)** Protocol for iPSC differentiation to EC. Panels a-d are based on scRNA-Seq dataset GSE116555; Panele is based on scRNA-Seq dataset GSE131736. Source data are provided as a Source Data file.

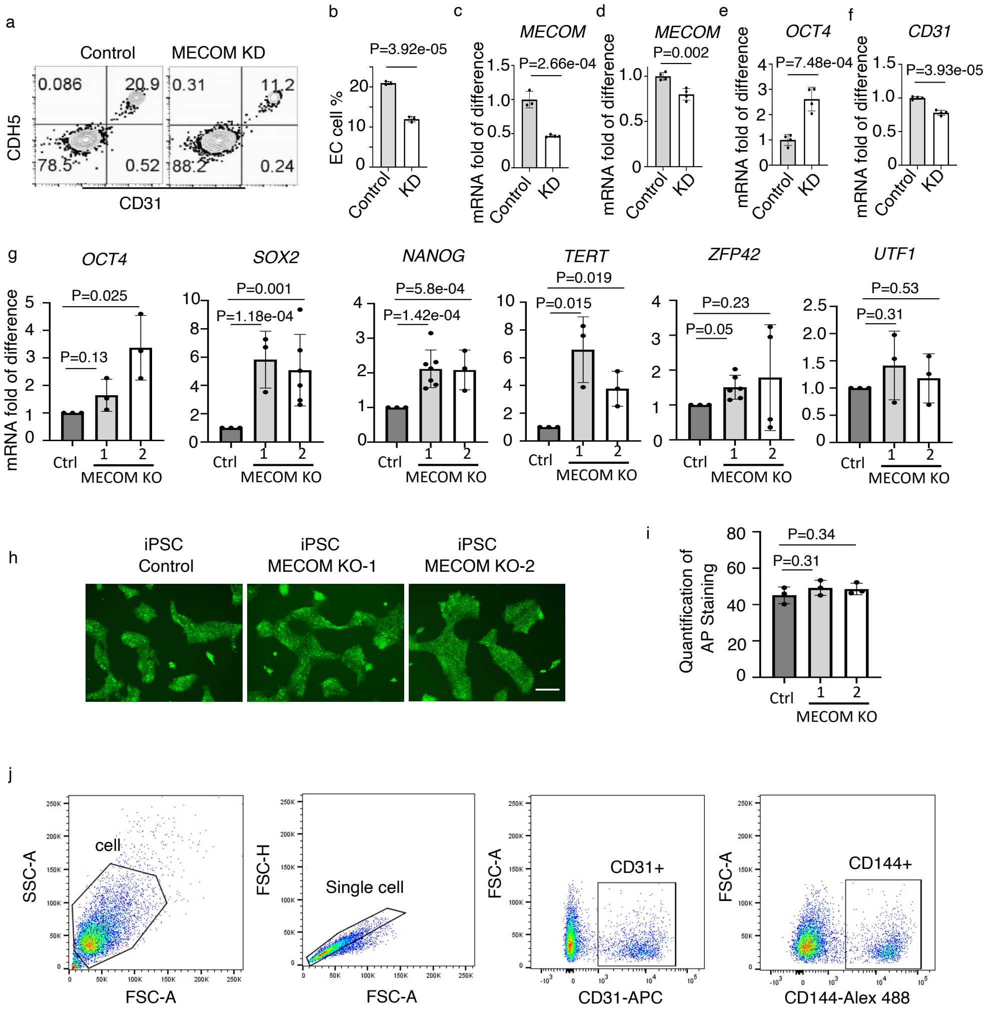

Figure S5 MECOM KD by siRNA impaired EC differentiation of iPSC. **(a)** FACS analysis of $CD31^+CDH5^+$ ECs at day 7. **(b)** Quantification of $CD31^+CDH5^+$ EC percentage at day 7. **(c-d)** *MECOM* expression levels at day 1 **(c)** and day 7 **(d)**. **(e-f)** Expression level of *OCT4* **(e)** and *CD31* **(f)** at day 7. **(g)** Relative gene expression levels of iPSC pluripotency marker. **(h)** Representative fluorescence images of Alkaline Phosphatase (AP) live staining of iPSC. Scale bar, 200 μm. **(i)** Quantification of AP live staining in figure S5h. (**j**) Gating strategy for FACS analysis. Mean ± SE; n=3 biologically independent samples (**a-i**). P values are determined by two-tailed Student's t-test. Source data are provided as a Source Data file.

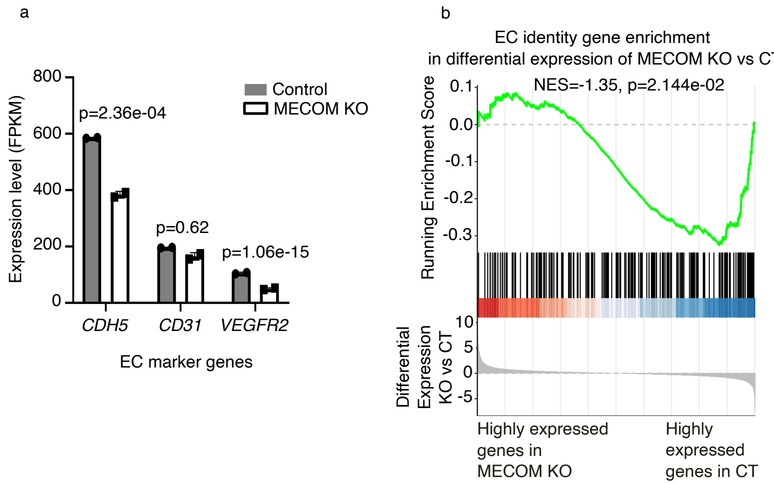

Figure S6 Loss of EC identity upon knocking out MECOM. (a) mRNA expression level in FPKM of EC markers including *CDH5*, *CD31*, and *VEGFR2* in *MECOM* wild type and knock out cells. The p values were calculated by edgeR package in R. **(b)** Gene Set Enrichment Analysis (GSEA) of EC identity genes in *MECOM* knock out EC samples compared to wild type control. The normalized enrichment score (NES) and p value were calculated by fgsea package in R. Data are presented as mean values +/- SD (**a**). n=2 biologically independent samples (**a**). P values were determined by two-tailed Negative Binomial test implemented in edgeR v3.14 (**a**) and permutation test (**b**). Source data are provided as a Source Data file.

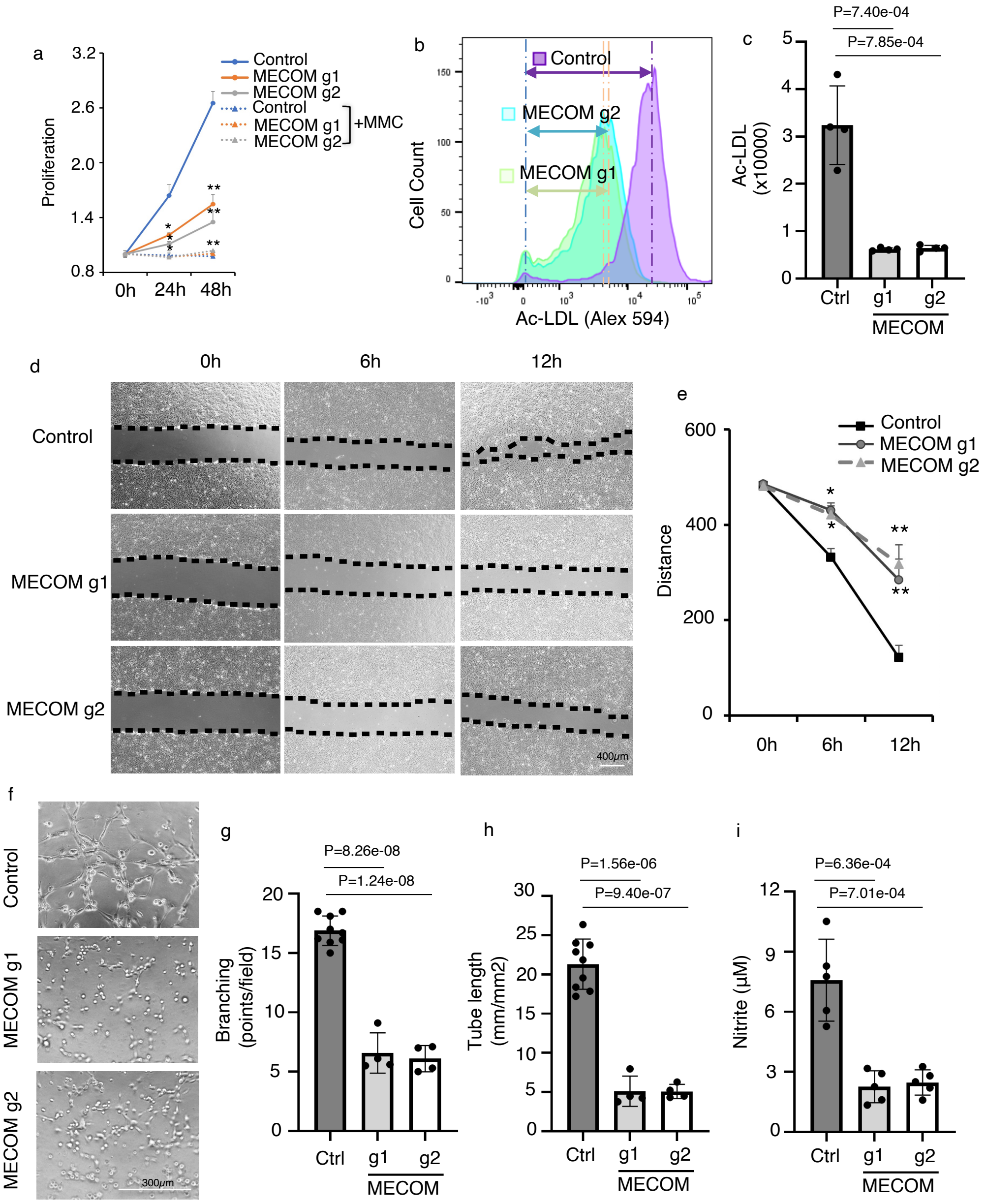

Figure S7 MECOM is required for HUVEC phenotype and function. (a) HUVEC proliferation. **(b)** LDL uptake measured by flow cytometry. **(c)** Quantification of LDL uptake. **(d)** Representative wound closure images at 0h, 6h, and 12h after scratch. **(e)** Distance between the two borders (dotted lines in **d**) in wound closure images. **(f)** Representative images of in vitro tube formation. **(g)** The number of EC tube branching points per field. **(h)** Quantification of tube length. **(i)** Nitrite production by HUVECs. Two CRISPR gRNA were tested. Data are presented as mean values +/- SD (**a,c,e,and g-i**). n=3 biologically independent samples (**a-i**). P values were determined by two-tailed Student's T-test. *P <0.05, **P <0.01. Source data are provided as a Source Data file.

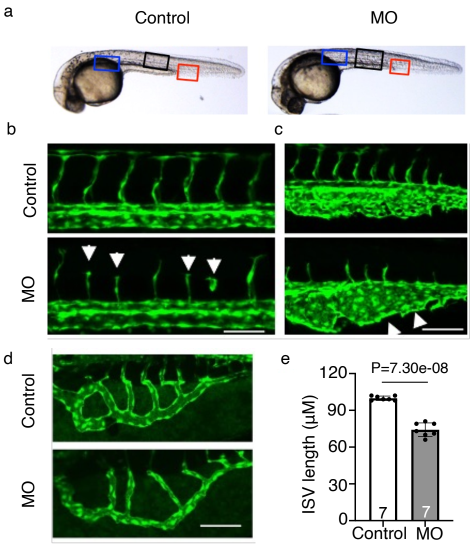

Figure S8 MO-mediated loss of *mecom* impairs zebrafish blood vessel formation. **(a)** No gross defects in *mecom* MO embryos at 24 hpf. The black, red and blue rectangles correspond to imaging area in **(b)**, **(c)**, and **(d)** respectively. *mecom* knockdown retarded ISV angiogenesis (**b**) and disrupted venous plexus formation (**c**) but did not affect SIV formation (**d**). Arrows in **(b)** indicate impaired ISV vessels. Arrowheads in **(c)** show restricted venous angiogenesis. **(e)** Quantified ISV length in **(b)**. Scale bar, 100 um. Mean ± SE; P values were determined by two-tailed Student's t test. Numbers of animals are indicated in bar plots (**a-e**). Source data are provided as a Source Data file.

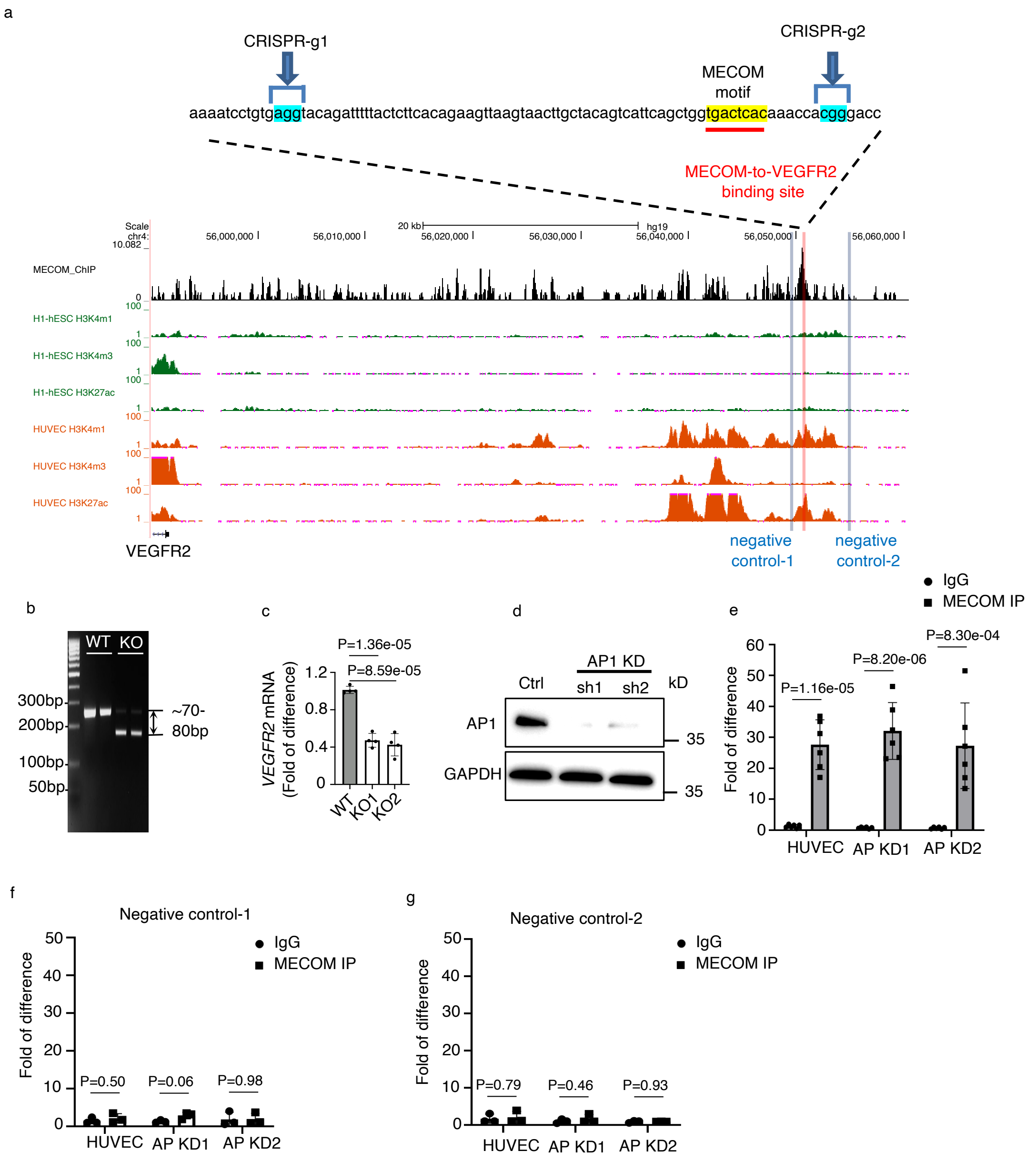

Figure S9 VEGF signaling pathway is a key target of MECOM. (**a**) Location and epigenetic landscape of inferred MECOM-to-VEGFR2 binding site (red line) and two negative control sites for MECOM binding (blue line). The enlarged figure shows the location of MECOM binding motif and the two CRISPR gRNAs. (**b**) HUVEC cells were infected by lentiviral gRNA1 and gRNA2. The CRISPR targeted MECOM-to-VEGFR2 binding site locus from WT and infected HUVECs were PCR amplified and size-separated by electrophoresis. (**c**) mRNA levels of *VEGFR2* determined by qRT-PCR. (**d**) Western blot analysis of protein levels of AP1 and GAPDH in wild type and AP1 KD HUVEC. (**e-g**) MECOM ChIP-PCR with IgG as control in both wild type and AP-1 KD HUVECs for MECOM-to-VEGFR2 binding site (**e**) and two negative control sites (**f-g**). Data are presented as mean values +/- SD (**c,e-g**). n=3 biologically independent samples (**b-g**). P values were determined by two-tailed Student's test. Source data are provided as a Source Data file.
