# Supplementary material for: Epigenetic landscape reveals MECOM as an endothelial lineage regulator
Source: Nat Commun. 2023 Apr 25;14:2390. doi: 10.1038/s41467-023-38002-w (PMC10130150; doi:10.1038/s41467-023-38002-w)
Supplement: Supplementary file 3 — Description of Additional Supplementary Files [file 41467_2023_38002_MOESM3_ESM.pdf]

Description of Additional Supplementary Files

File Name: Supplementary Data 1
Description: Pearson correlation coefficients among histone modifications.

File Name: Supplementary Data 2
Description: 206 positive EC regulators defined by the intersection between the genes in the GO pathways that each have the keyword endothelial in the pathway name and the genes upregulated in EC compared to ESC. The p-value was calculated by the Cuffdiff function in Cufflink suite v2.2.1.

File Name: Supplementary Data 3
Description: 71 transcription factors in the gene regulation network defined for ECs by the algorithm CellNet.

File Name: Supplementary Data 4
Description: Database information for all datasets analyzed in this project.
