# Supplementary material for: Epigenetic landscape reveals MECOM as an endothelial lineage regulator
Source: Nat Commun. 2023 Apr 25;14:2390. doi: 10.1038/s41467-023-38002-w (PMC10130150; doi:10.1038/s41467-023-38002-w)
Supplement: Supplementary file 8 — Reporting Summary [file 41467_2023_38002_MOESM8_ESM.pdf]

nature portfolio

Corresponding author(s): Lili Zhang, Kaifu Chen, Longhou Fang, John P. Cooke

Last updated by author(s): Mar 26, 2023

Reporting Summary

Nature Portfolio wishes to improve the reproducibility of the work that we publish. This form provides structure for consistency and transparency in reporting. For further information on Nature Portfolio policies, see our Editorial Policies and the Editorial Policy Checklist.

Statistics

For all statistical analyses, confirm that the following items are present in the figure legend, table legend, main text, or Methods section.

n/a	Confirmed	
☐	☒	The exact sample size (n) for each experimental group/condition, given as a discrete number and unit of measurement
☐	☒	A statement on whether measurements were taken from distinct samples or whether the same sample was measured repeatedly
☐	☒	The statistical test(s) used AND whether they are one- or two-sided<br>*Only common tests should be described solely by name; describe more complex techniques in the Methods section.*
☒	☐	A description of all covariates tested
☒	☐	A description of any assumptions or corrections, such as tests of normality and adjustment for multiple comparisons
☐	☒	A full description of the statistical parameters including central tendency (e.g. means) or other basic estimates (e.g. regression coefficient) AND variation (e.g. standard deviation) or associated estimates of uncertainty (e.g. confidence intervals)
☐	☒	For null hypothesis testing, the test statistic (e.g. F, t, r) with confidence intervals, effect sizes, degrees of freedom and P value noted<br>*Give P values as exact values whenever suitable.*
☒	☐	For Bayesian analysis, information on the choice of priors and Markov chain Monte Carlo settings
☒	☐	For hierarchical and complex designs, identification of the appropriate level for tests and full reporting of outcomes
☐	☒	Estimates of effect sizes (e.g. Cohen's d, Pearson's r), indicating how they were calculated

Our web collection on statistics for biologists contains articles on many of the points above.

Software and code

Policy information about availability of computer code

Data collection	No software was used for data collection.
Data analysis	ChIP-seq and DNase-seq analyses were performed with Bowtie v1.1.0, DANPOS v2.2.3 and Multiple Experiment Viewer (MeV) v10.2. RNA-Seq analyses were performed with TopHat v2.0.12, Cufflink suite v2.2.1, edgeR v3.14.0, bedtools v2.25.0, bedGraphToBigWig v4, and Genomics Viewer (IGV) v2.3.67. Hi-C data analyses were processed using Juicebox v1.11.08 and juicer_tools v1.23.03. scRNA-seq data were processed with Seurat v2.0 installed on R version 3.5.1. Gene Ontology pathway analyses were performed with DAVID v6.8. Motif analyses were performed with HOMER v4.10. ChIP-Seq peaks and motifs of transcription factors (TFs) are assigned to target genes using Cumulative Analysis of Genomic Region Enrichment (CAGRE) v1.0 (https://github.com/jielv/CAGRE). Gene Regulatory Network (GRN) analysis is performed using custom scripts (https://github.com/jielv/GRN_analysis). Two-tailed Wilcoxon test, Fisher Exact test, and Student's t-Test are performed using R v4.0.2.

For manuscripts utilizing custom algorithms or software that are central to the research but not yet described in published literature, software must be made available to editors and reviewers. We strongly encourage code deposition in a community repository (e.g. GitHub). See the Nature Portfolio guidelines for submitting code & software for further information.

Data

Policy information about availability of data

All manuscripts must include a data availability statement. This statement should provide the following information, where applicable:

- Accession codes, unique identifiers, or web links for publicly available datasets
- A description of any restrictions on data availability
- For clinical datasets or third party data, please ensure that the statement adheres to our policy

The RNA-Seq datasets for wild type HUVECs and HUVECs with MECOM depleted by CRISPR-Cas9 were generated in this project and deposited to the GEO database by the accession number GSE160647. Other genomic datasets were downloaded from public database. Database accession numbers for all datasets analyzed in this project were indicated in Supplementary 4. Source data are provided with this paper.

Field-specific reporting

Please select the one below that is the best fit for your research. If you are not sure, read the appropriate sections before making your selection.

☒ Life sciences ☐ Behavioural & social sciences ☐ Ecological, evolutionary & environmental sciences

For a reference copy of the document with all sections, see nature.com/documents/nr-reporting-summary-flat.pdf

Life sciences study design

All studies must disclose on these points even when the disclosure is negative.

Sample size	For cellular experiment, 3-6 individual samples were used in each group. Sample size were determined based on our previous experience and similar studies of other groups. For Zebrafish experiment, 5-35 individual samples were used in each group. Sample size were determined to satisfy the significance or statistical requirement, similar to other groups in zebrafish community.<br><br>Reference:<br>Shull LC, Sen R, Menzel J, Goyama S, Kurokawa M, Artinger KB. The conserved and divergent roles of Prdm3 and Prdm16 in zebrafish and mouse craniofacial development. Dev Biol. 2020; 461:132-144.
Data exclusions	No data were excluded.
Replication	All experiments have been repeated for at least 3 times. All experiments were reproduced to reliably support conclusions stated in the manuscript.
Randomization	Cells in different group were passaged from the same cell line and randomly assigned to experimental groups. For the zebrafish experiment, samples were allocated to experimental groups based on their genotype or transgenic line.
Blinding	After treatment, different groups were labeled with numbers only. During the data collection and analysis, the investigators were blinded to group allocation.

Reporting for specific materials, systems and methods

We require information from authors about some types of materials, experimental systems and methods used in many studies. Here, indicate whether each material, system or method listed is relevant to your study. If you are not sure if a list item applies to your research, read the appropriate section before selecting a response.

Materials & experimental systems

n/a	Involved in the study	
☐	☒	Antibodies
☐	☒	Eukaryotic cell lines
☒	☐	Palaeontology and archaeology
☐	☒	Animals and other organisms
☒	☐	Human research participants
☒	☐	Clinical data
☒	☐	Dual use research of concern

Methods

n/a	Involved in the study	
☒	☐	ChIP-seq
☐	☒	Flow cytometry
☒	☐	MRI-based neuroimaging

Antibodies

Antibodies used	All antibodies are commercially available and have been tested for the species used in this manuscript. Following antibodies were used:

1. CD31 (PECAM-1) Monoclonal Antibody (WM-59 (WM59)), APC, eBioscience™, Catalog # 17-0319-41, Thermo Scientific. 1:100 dilution in FACSB-10 buffer.
2. CD144 (VE-cadherin/CDH5) Monoclonal Antibody (16B1), Alexa Fluor 488, eBioscience™, Catalog # 53-1449-41, Thermo Scientific. 1:100 dilution in FACSB-10 buffer.
3. EVI-1(C50E12) Rabbit Monoclonal Antibody, Catalog # 2593, Cell Signaling Technology, Inc. 1:500 dilution in PBST.
4. Anti-KDR/VEGFR2 mouse Monoclonal Antibody (A-3), Catalog# sc-6251, SANTA CRUZ BIOTECHNOLOGY, INC. 1:500 dilution in PBST .
5. GAPDH mouse monoclonal antibody (6C5), Catalog #sc-32233, SANTA CRUZ BIOTECHNOLOGY, INC. 1:1000 dilution in PBST.
6. Anti-α Tubulin mouse monoclonal antibody (B-7), Catalog #sc-5286, SANTA CRUZ BIOTECHNOLOGY, INC. 1:1000 dilution in PBST.
7. β-Actin Antibody, Cat#: 4967, Cell Signaling Technology, Inc. 1:1000 dilution in PBST.
8. Peroxidase AffiniPure Goat Anti-Rabbit IgG (H+L), cat# 111-035-144, Jackson ImmunoResearch Laboratories INC. 1:5000 dilution in PBST.
9. c-Jun (AP1, 60A8) Rabbit mAb, Cat #9165, Cell Signaling Technology, Inc. 1:1000 dilution in PBST.

Validation

Antibodies we used were chosen based on the published work in the field. Each antibody was validated by Western blot (correct size of the detected bands based on the protein marker) as shown in the figure of this manuscript. Proper controls were also used for validation.

Eukaryotic cell lines

Policy information about cell lines

Cell line source(s)

Human Umbilical Vein Endothelial Cells (HUVEC) were purchased from Lonza (#: CC-2517). hPSC (Human pluripotent stem cells) was a kind gift from Dr. John Cooke's group. HEK293T cell were purchased from ATCC (#CRL-3216).

Authentication

Genotyping followed by Sanger sequencing, followed by qPCR or Western blot

Mycoplasma contamination

All these cells were mycoplasma negative during routine tests

Commonly misidentified lines
(See ICLAC register)

No

Animals and other organisms

Policy information about studies involving animals; ARRIVE guidelines recommended for reporting animal research

Laboratory animals

6 months adult transgenic zebrafish line Tg(fil1a:EGFP)y1, male: 20, female: 20. All adult fish are used to produce embryos for research and breeding. All zebrafish larvae before 5 day post fertilization are used for assays.

Wild animals

No use of wild animals

Field-collected samples

No use of field-collected samples

Ethics oversight

All the animal studies were approved by the Houston Methodist Institution Animal Care Use Committee.

Note that full information on the approval of the study protocol must also be provided in the manuscript.

Flow Cytometry

Plots

Confirm that:

☒ The axis labels state the marker and fluorochrome used (e.g. CD4-FITC).

☒ The axis scales are clearly visible. Include numbers along axes only for bottom left plot of group (a 'group' is an analysis of identical markers).

☒ All plots are contour plots with outliers or pseudocolor plots.

☒ A numerical value for number of cells or percentage (with statistics) is provided.

Methodology

Sample preparation

7-10 days after differentiation, iPSCs were trypsinised, centrifuged at 200 x g for 5min, resuspended in FACSB-10 (FACS buffer–10% FBS) and incubated with anti human-CDH5 and anti human CD31 for 30 min on ice

Instrument

Fluorescence was determined using a flow cytometer (LSR II, Becton Dickinson, San Jose, CA, USA)

Software

The data were analyzed using FlowJo software.

Cell population abundance

Purity of post-sort fractions is regularly measured by flow cytometry core facility and >90%

Gating strategy

Single cells are identified by plotting forward scatter-area against forward scatter-height. single cells are then separated from

Gating strategy

debris by a forward versus side scatter. CD31+CDH5+ cells were defined as induced endothelial cells.

☒ Tick this box to confirm that a figure exemplifying the gating strategy is provided in the Supplementary Information.
